# Supplementary material for: Content-rich biological network constructed by mining PubMed abstracts
Source: BMC Bioinformatics. 2004 Oct 8;5:147. doi: 10.1186/1471-2105-5-147 (PMC528731; doi:10.1186/1471-2105-5-147)
Supplement: Additional File 2 — The original results of the above study (non-essential files are deleted to keep the file size under the limit set by BMC bioinformatics). [file 1471-2105-5-147-S2.bz2 › chilibotAdditionalFile2/dip05/286ID9571155E1111/html/BMI1_CBX2.html]

 


 **BMI1** and **CBX2** 
  
Found 12 abstracts in PubMed, retrieved 05.  
 

 What does Google say? 
 PDF only 
| .edu only 

---

**Interactive relationship** (e.g. stimulation, inhibition, etc)

**Non-interactive relationship** (e.g. studied together, co-existance, homology, etc.)

- Four mammalian homologues, mel 18, bmi 1  [ **BMI1** ] , M33  [ **CBX2** ]  and rae 28, are analyzed in this study.  Ref: 9809583 Mol Immunol, 1998
- Here we show that this PEV is influenced by the heterochromatic protein SUV39H1 and by the Polycomb group proteins M33  [ **CBX2** ]  and BMI 1  [ **BMI1** ] .  Ref: 10990462 EMBO J, 2000
- Mice deficient for homologues of the Drosophila PcG genes, such as M33  [ **CBX2** ] ,  **bmi1** , mel18, rae28 and eed, show altered Hox expression patterns.  Ref: 11044623 Mech Dev, 2000
